# Sperm-Derived Dysfunction of Human Embryos: Molecular Mechanisms and Clinical Resolution

**DOI:** 10.3390/ijms26136217

**Published:** 2025-06-27

**Authors:** Jan Tesarik, Raquel Mendoza Tesarik

**Affiliations:** MARGen (Molecular Assisted Reproductiona and Genetics) Clinic, 18006 Granada, Spain; clinicamargen@gmail.com

**Keywords:** sperm factor, embryo development, oocyte activation, centriole, DNA, RNA, diagnosis, treatment

## Abstract

In addition to the male genome, the fertilizing spermatozoon delivers to the oocyte several factors whose deficiency can cause embryo dysfunction. Sperm oocyte-activating factor, identified as phoshoplipase C zeta (PLCζ), drives oocyte exit from meiotic arrest through a signaling pathway initiated by periodic rises of free cytosolic Ca^2+^ concentration (calcium oscillations). Sperm centrioles, together with oocyte proteins, form centrosomes that are responsible for aster formation, pronuclear migration, and DNA polarization before nuclear syngamy and subsequent mitotic divisions. Sperm DNA fragmentation can be at the origin of aneuploidies, while epigenetic issues, mainly abnormal methylation of DNA-associated histones, cause asynchronies of zygotic gene activation among embryonic cells. Sperm long and short non-coding RNAs are important epigenetic regulators affecting critical developmental processes. Dysfunction of sperm PLCζ, centrioles, DNA, and RNA mostly converge to aneuploidy, developmental arrest, implantation failure, miscarriage, abortion, or offspring disease. With the exception of DNA fragmentation, the other sperm issues are more difficult to diagnose. Specific tests, including heterologous human intracytoplasmic sperm injection (ICSI) into animal oocytes, genetic testing for mutations in *PLCZ1* (the gene coding for PLCζ in humans) and associated genes, and next-generation sequencing of sperm transcriptome, are currently available. Oral antioxidant treatment and in vitro selection of healthy spermatozoa can be used in cases of sperm DNA fragmentation, while ICSI with assisted oocyte activation is useful to overcome oocyte-activation defects. No clinically confirmed therapy is yet available for sperm RNA issues.

## 1. Introduction

Since in vitro fertilization (IVF) became one of the most popular treatments for human infertility caused by various issues of gamete quality, many technical advances in IVF techniques have been achieved. Despite that, the clinical efficacy of IVF has not kept pace with these achievements and still remains disturbingly low [1]. There is a strong consensus among experts that this drawback is mainly associated with embryo quality and derives from impaired function of gametes. Historically, IVF was developed for female infertility of tubal origin to bypass the obstacle impeding spermatozoa and oocytes from meeting [2]. However, it was rapidly understood that IVF can also be used with success in male infertility with diminished sperm count and motility so as to facilitate sperm access to the oocyte by avoiding physiological barriers present in the upper female genital tract [3]. The development of laboratory techniques to assist fertilization by means of micromanipulation, mainly intracytoplasmic sperm injection (ICSI) [4], shifted the balance between the female and male indications of IVF in favor of the latter [5], and this is even more the case with round spermatid injection (ROSI) [6] which still remains substantially inferior to standard ICSI with mature spermatozoa according a recent meta analysis [7].

Oocytes were traditionally blamed for IVF success rates still remaining far below the desired ones. This was reasonably true for steadily growing IVF indications in women of advanced age. Embryo demise in other cases was usually explained by “hidden” oocyte issues. However, as early as the first decade of this century, sperm quality was shown to be implicated in dysfunction of human embryos from the very early stages of development, even before activation of the embryonic genome [8]. However, it is only recently that the potential sperm origin of embryo dysfunction has been increasingly acknowledged and demonstrated by clinical studies [1,9,10]. Consequences of this dysfunction include preimplantation developmental arrest [9,11], implantation failure [12], miscarriage [13,14], and offspring abnormalities [15]. The use of micromanipulation techniques to assist fertilization [4,5,6,7] has been shown to act as an additional contributing factor by further limiting natural mechanisms of sperm selection as compared with conventional IVF [16].

This review deals with the principal sperm-derived factors whose dysfunction can cause human embryo developmental arrest, implantation failure, miscarriage, abortion, and offspring abnormalities. The presentation of each factor is structured so as to provide biologists and clinicians with an armory of knowledge required for research outlook and comprehensive clinical management. With this aim, molecular mechanisms of action and the consequences of their deficiency are presented first, followed by the outline of currently available diagnostic methods and treatment options.

## 2. Sperm Oocyte-Activating Factor

### 2.1. Molecular Basis

At the outset of fertilization, the cell cycle of the oocyte is arrested at the metaphase of the second meiotic division. In order to complete meiosis and start the first embryonic cell cycle, the oocyte needs to be activated by the fertilizing spermatozoon. Oocyte activation is initiated by calcium signals elicited by a factor released from the spermatozoon. The sperm oocyte-activating factor has been identified as a special form of phospholipase C (PLC) referred to as PLC zeta (PLCζ). It was first discovered in mouse [17] and later in human spermatozoa where it was localized to the equatorial region [18], more precisely along the inner acrosomal membrane and in the perinuclear theca [19], and it was undetectable in sperm from some patients with a history of failed ICSI [18,19].

**Figure 1:**
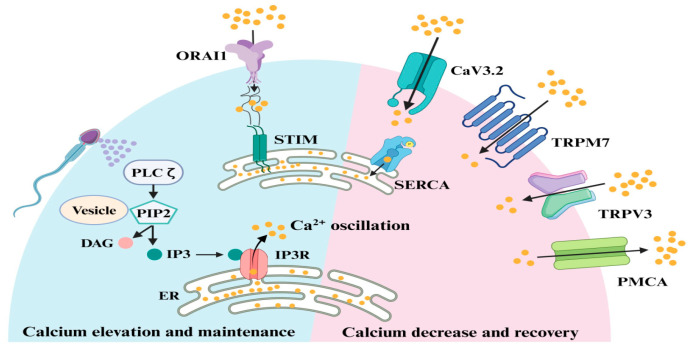
Sperm PLCζ hydrolyzes phosphatidylinositol 4,5-bisphosphate (PIP2) to generate inositol trisphosphase (IP3), which is required for Ca^2+^ release from oocyte IP3-sensitive stores, mainly endoplasmic reticulum (ER) to the cytosol. Once the ER store is emptied, the oocyte replenishes the depleted ER store by influx of extracellular Ca^2+^ through store-operated Ca^2+^ entry mediated by stromal interaction molecule (STIM) proteins and ORAI (word derived from Greek mythology) Ca^2+^ channels. In response to a reduction in ER Ca^2+^, the STIM proteins interact directly with ORAI channels, inducing Ca^2+^ influx. This Ca^2+^ is subsequently pumped back into the ER by the action of sarco-ER Ca^2+^ ATPases (SERCA). The overall stability of intracellular Ca^2+^ is controlled by voltage-gated (CaV3.2) and transient receptor potential (TRPM7, TRPV3) channels as well as P-type Ca^2+^ pumps (PMCA). From Chen et al. [20]. ©2024 Chen, Huang, Dong, Ding, Li, Wang, Zeng, Zhang and Sun. Creative Commons Attribution License.

The mechanism of sperm-induced calcium signals in oocytes (Figure 1) has been nicely reviewed recently [20].

Deficiency of PLCζ is sometimes associated with sperm morphology. This is mainly the case of globozoospermia, a condition characterized by a high percentage of round-headed spermatozoa within a sperm sample; such spermatozoa lack the acrosome and present an aberrant nuclear membrane and midpiece defects [21]. Globozoospermia occurs in less than 0.1% of infertile patients worldwide [22]. While the absence of acrosome hinders natural fertilization and conventional IVF, the lack of oocyte activation through the action of PLCζ is a major problem in ICSI. There are various forms of globozoospermia with different prognostic phenotypes; they differ both in the percentage of affected sperm cells and their oocyte-activating ability [23]. On the other hand, normal-appearing spermatozoa can also be deficient in PLCζ at the magnifications used in standard sperm morphology analysis [24], and delving into the real value of sperm morphology in predicting fertilization and embryo development outcomes still remains a challenge.

**Figure 2:**
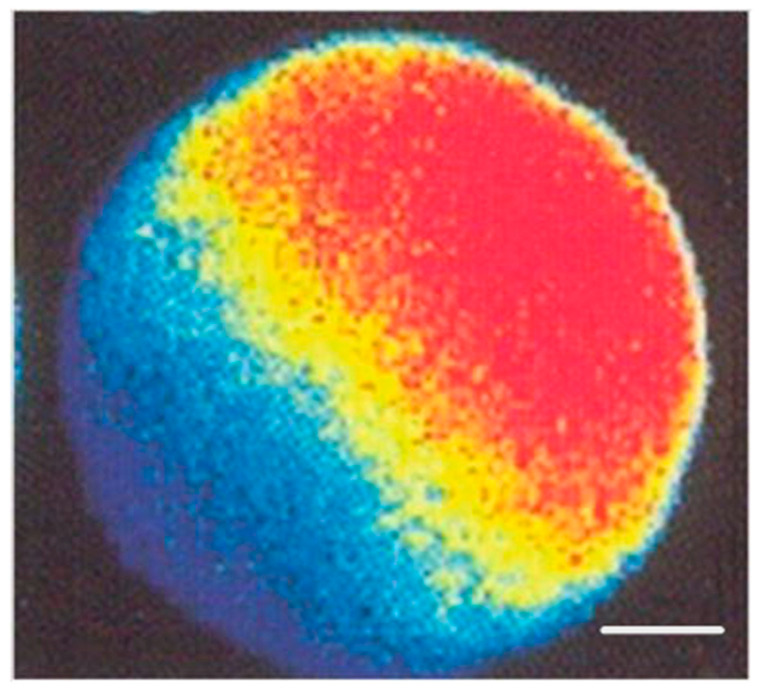
Confocal microscopy snapshot of a human oocyte, taken several seconds after sperm/oocyte fusion. The oocyte was previously loaded with fluorescent Ca^2+^ indicator Fluo-3. Fluorescence intensity, directly proportional to local Ca^2+^ concentration, is converted to pseudocolor, going from the highest (red) to the lowest (dark blue). Zone of increased cytosolic free Ca^2+^ concentration is in progress, running from the site of sperm/oocyte fusion (upper right) across the whole cytoplasm towards the opposite pole. Scale bar = 25 μm. From Tesarik [26]. © 2019 Jan Tesarik. Creative Commons Attribution License. Tesarik. Creative Commons Attribution License.

Two different types of Ca^2+^ stores, the inositol trisphosphate (IP3)-sensitive ones and the ryanodine-sensitive ones, are involved in sperm-induced calcium signaling [25,26,27]. The IP3-sensitive Ca^2+^ stores are accumulated in the peripheral region of the oocyte from which the signal (Ca^2+^ wave) runs across the whole oocyte (Figure 2) owing to Ca^2+^-induced Ca^2+^ release from ryanodine-sensitive stores that are spread all over the bulk of the oocyte cytoplasm [25,26].

**Figure 3:**
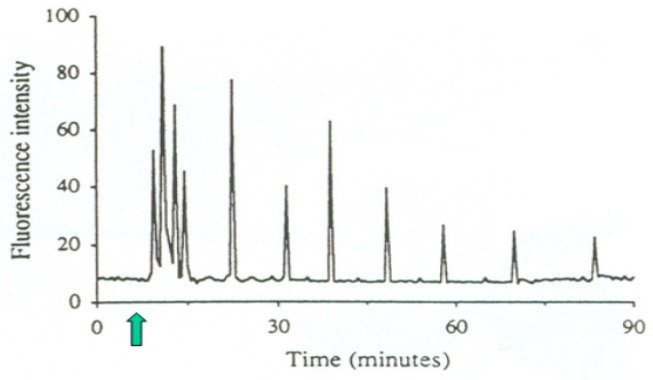
Oscillations of free cytosolic Ca^2+^ concentration induced by sperm/oocyte fusion (arrow), recorded by confocal microscopy in a living human oocyte loaded with fluorescent Ca^2+^ indicator Fluo-3. Periodic increases in fluorescence intensity reflect those in cytosolic Ca^2+^ concentration. From Tesarik [26]. © 2019 Jan Tesarik. Creative Commons Attribution License.

In addition to this particular spatial pattern, the sperm-induced calcium signal also has a specific temporal pattern (Figure 3), characterized by a series of periodic sharp increases and decreases of cytosolic free Ca^2+^ concentration, termed calcium oscillations [26].

Apart from causing complete fertilization failure, minor deficiencies of PLCζ can sometimes be compatible with fertilization but cause persistent embryo quality issues and/or miscarriage after embryo transfer [26]. Specifically, abnormalities of sperm-induced calcium signals can cause complete failure of the second meiotic division, leading to triploidy; incomplete failure of the second meiotic division, leading to de novo chromosomal numerical abnormalities; abnormal pronuclear development and function; abnormalities of the blastomere cell cycle, possibly leading to embryo cleavage arrest; and problems with blastomere allocation to embryonic cell lineages, leading to disproportionate development of the inner cell mass and trophectoderm derivatives, which can be the origin of implantation failure or miscarriage [27]. More recently, a homozygous missense mutation of the actin-like 7A (*ACTL7A*) gene was identified by whole-exome sequencing in two infertile brothers, and a corresponding mutated mouse model was generated [28]. Both the infertile brothers and the model mice exhibited reduced expression of PLCζ in spermatozoa and a complete fertilization failure after ICSI, which could be overcome by assisted oocyte activation (see Section 2.2.2. of this article for methodological details). It has to be underscored that the mutation was undetectable by conventional semen analysis and that the individuals were homozygous for it [28]. In cases of heterozygous mutations, a slight reduction of PLCζ can be expected, leading to early embryo dysfunction rather than complete fertilization failure. In fact, changes in the expression or intracellular position of PLCζ in spermatozoa are associated with subfertility or even infertility owing to impaired embryonic development [29], and ACTL7A protein levels were shown to be significantly reduced in sperm samples presenting poor embryo quality [30].

Later studies revealed other genetic causes of sperm-related human infertility, including homozygous pathogenic variants in the actin-like 9 (*ACTL9)* gene [31], disruption of the IQ motif-containing N (*IQCN*) gene [32], and bi-allelic mutations in *PLCZ1* (the gene coding for PLCζ in humans) [33,34].

### 2.2. Clinical Resolution

#### 2.2.1. Diagnostic Methods

Serious problems of the sperm oocyte-activating factor can be easily blamed in cases of total fertilization failure after ICSI. However, slighter forms of deficiency, leading to impaired embryo development after apparently normal fertilization, can only be detected by evaluating sperm-induced oscillations by confocal microscopy after oocyte loading with intracellular calcium indicators [35,36], which is incompatible with oocyte survival and further embryo development. In order to obviate this problem, tests substituting human oocytes with animal ones were developed. Thus far, mouse oocytes are the most commonly used model with which to study the oocyte-activating capacity of human sperm because of their ease of access and handling, high cleavage rate after intracytoplasmic injection of human sperm, and the relatively low rate of spontaneous activation [37]. Piezo-driven ICSI of human sperm into mouse oocytes can be used both to assess the activation rate (mouse oocyte activation test, MOAT) [38,39] and the sperm-induced calcium oscillation pattern (mouse oocyte calcium analysis, MOCA) [40]. The performance of patients’ spermatozoa is compared to that of spermatozoa from fertile donors in both of these tests. The MOCA test is particularly useful in patients whose oocytes do undergo fertilization after ICSI but subsequently develop into dysfunctional embryos to evaluate the relative contribution of spermatozoa and oocytes to this condition. In addition to mouse, heterologous ICSI with hamster oocytes was also used to assess sperm oocyte-activating performance [41].

In some cases, defective function of sperm oocyte-activating factor can be detected by direct observation of sperm cells. This is easy in patients with globozoospermia, the most notorious anomaly associated with the inability of spermatozoa to activate oocytes (see Section 2.1 of this article). Spermatozoa from these patients lack an acrosome and show a deficiency of the oocyte-activating factor PLCζ, making them unable to correctly activate oocytes even when injected into their cytoplasm by ICSI [42]. However, in most men, the insufficiency of sperm oocyte-activating factor is associated with subtler phenotypical manifestations that cannot be distinguished by conventional semen analysis. This is the case of disruption in actin-like 7A (*ACTL7A*) that is associated with acrosomal defects detectable by cytochemistry and electron microscopy [28]. Reduced sperm ACTL7A protein levels were shown to be significantly associated with poor embryo quality and suggested as a biomarker for assisted reproductive technology outcomes [30]. Genetic testing for *PLCZ1* (the gene coding for PLCζ in humans) mutations [33,34] can also be envisaged when issues of sperm-induced oocyte activation are suspected.

#### 2.2.2. Treatment Options

Absent or reduced ability of spermatozoa to activate the oocyte, leading, respectively, to fertilization failure and impaired embryo development, can be treated with success by assisted oocyte activation (AOA) after ICSI. Even when performed as late as 24 h after ICSI, most of the oocytes that initially failed to fertilize did so after subsequent AOA by exposure to calcium ionophore and underwent at least one apparently normal cleavage division [35]. The beneficial effect of AOA was substantiated by the demonstration that unfertilized sperm-injected oocytes subjected to AOA with the use of calcium ionophore A23187 (calcimycin) developed free cytosolic Ca^2+^ oscillations, quite similar to those observed after sperm/oocyte fusion [36]. This oocyte response to ionophore only occurred when a spermatozoon or a round spermatid (haploid sperm precursor cell) was present in their cytoplasm, and treatment of oocytes previously sham-injected with non-germ cells (leukocytes) merely displayed a single transient Ca^2+^ rise [36]. Nowadays, there are many reports on the use of AOA with calcimycin, and all of them agree that the method is efficient in improving fertilization and embryo development after ICSI in couples with previous problems, even in those in which the implication of sperm oocyte-activating factor has not been clearly ascertained [33,39,42,43,44,45,46,47,48,49]. In addition to calcium ionophores, successful chemical-free activation of human oocytes can also be achieved by a special ICSI technique (double vigorous cytoplasmic aspiration) [50] or exposure of oocytes to an electrical field [51].

The safety of AOA with calcium ionophores was assessed in several studies that addressed the frequency of chromosome segregation errors in the second meiotic division of the sperm-injected oocytes [52], and neonatal and neurodevelopmental outcomes of children born after uterine transfer of the resulting embryos [53,54,55,56]. None of these studies evidenced any serious adverse effects in any of these aspects. Even so, it has to be admitted that, in spite of these encouraging results, the sample sizes of these studies are relatively low, and more follow-up evaluations of children born after AOA are required. For the time being, in order to avoid any trace of doubt concerning the use of calcium ionophores, the recourse to drug-free AOA (see above) is possible.

## 3. Sperm Centrioles

### 3.1. Molecular Basis

**Figure 4:**
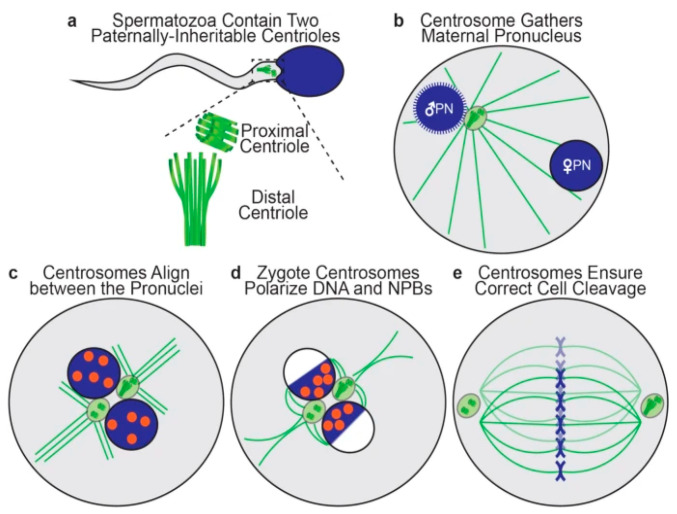
Schematic representation of zygote centriole dynamics: (**a**) Two centrioles in the sperm neck. (**b**) The zygote with a male and a female pronucleus (PN), two centrioles (green circle) and microtubule aster (green lines). (**c**) The two pronuclei are in apposition with nucleolar precursor bodies (orange) and divided centrioles forming centrosomes. (**d**) The two centrosomes polarize DNA with nucleolar precursor bodies (NPBs) toward the interpronuclear contact zone. (**e**) The two centrosomes associate with the dual spindles poles, helping to organize and ensure correct cell cleavage. From Kluczynski et al. [60]. ©2025 The authors. Creative Commons Attribution License.

Each human spermatozoon has two centrioles, while the oocyte has none [57]. The sperm centrioles (Figure 4a), a barrel-shaped (typical) proximal one and a fan-shaped (atypical) distal one, are both located in the sperm neck (a small region between sperm head and midpiece) and exert multiple functions during fertilization and subsequent embryo development [58]. The human zygote inherits both sperm centrioles [59]. The actions of sperm centrioles in the human zygote (Figure 4a–e) were resumed recently [60] and are highly dependent on the proper function of centriolar proteins, mainly centrin, α-tubulin, and γ-tubulin [61].

Shortly after sperm/oocyte fusion (or sperm injection into the oocyte), the paternal centrioles are close to each other and to the sperm nucleus (becoming the male pronucleus) (Figure 4b). They form the zygote’s first centrosome by recruiting pericentriolar proteins from the oocyte, and send out a microtubule aster (Figure 4b) to pull the maternal pronucleus towards the paternal one, leading to pronuclear apposition (Figure 4c). After replication, the two zygote centrosomes, each containing two sperm-derived centrioles, align in the interpronuclear area (Figure 4c), interact with pronuclei’s nuclear pores and attract DNA, visualized in living oocytes by microscopic observation of nucleolar precursor bodies (NPBs), toward the area of interpronuclear contact in preparation of the first cleavage division (Figure 4d). Subsequently, the two centrosomes associate with the dual spindles poles, helping to organize and ensure correct mitotic division during the first embryonic cleavage (Figure 4e) [60]. Both epidemiological and observational studies (reviewed by Avidor-Reiss et al., 2019) [57] strongly suggest that centriole abnormalities may be a cause of human embryo dysfunction and failure to carry pregnancy to term.

### 3.2. Clinical Resolution

#### 3.2.1. Diagnostic Methods

**Figure 5:**
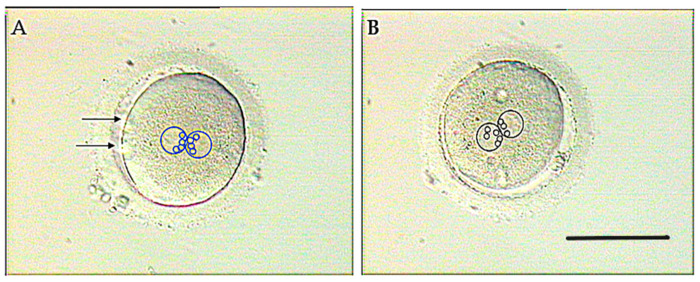
Distribution of NPBs, signaling the position of chromosomes, in the pronuclei of a normal (**A**) and an abnormal (**B**) zygote. Both the pronuclei and the NPBs are encircled to highlight their position. In the normal zygote, the NPBs (small blue circles) are symmetrically polarized in each pronucleus, accumulating in the interpronuclear contact region (**A**), while the NPBs (small black circles) polarization is lagging behind in one of the pronuclei (the left-side one) in the abnormal zygote (**B**). Arrows in (**A**) point to the polar bodies. Scale bar = 100 μm. Adapted from Tesarik 2025 [62]. Creative Commons Attribution Licence.

Defective function of sperm centrioles can be suspected when NPBs, marking de position of DNA in both pronuclei (see Section 3.1 of this article), fail to get adequately polarized in the zygote (Figure 5) [62].

The pattern of NPB polarization has significant correlations with the morphokinetic characteristics of cleaving embryos and euploidy [63]. However, abnormal pronuclear patterns are not exclusive to centriolar issues and can also be caused by oocyte-derived factors. For a more specific diagnosis of centriole dysfunction, recourse to heterologous ICSI can be made [37]. Given that mouse oocytes cannot be used for this objective as the embryonic centrosome is maternally derived in rodents [64,65], rabbit [66] and bovine [67,68] oocytes were used since the centrosome is paternally inherited in these species, just as in humans.

To analyze the functional competence of human sperm centrioles directly, Fluorescence-based Ratiometric Assessment of Centrioles (FRAC) was developed [69] and shown to be a robust, location-based, ratiometric assay of human centriole quality [60]. The finding of a lower centrosome protein expression in oligosthenozoospermic men as compared with normozoospermic ones [61] may serve as a basis for the development of rapid proteomic methods to predict sperm centriolar function.

#### 3.2.2. Treatment Options

As to the treatment of embryo dysfunction caused by defective function of sperm centrioles, very scarce data are available. Only one study addressed this subject in 2005, and it was found that zygote centrosomal function could be improved by the treatment of human spermatozoa with dithiothreitol before ICSI and of oocytes with paclitaxel after ICSI [70]. However, these pioneering observations have not been confirmed by any subsequent study so far.

## 4. Sperm DNA

### 4.1. Molecular Basis

There are multiple ways that factors affecting sperm DNA can influence early embryonic development, even before the major activation of embryonic gene transcription [71] and expression [72,73], taking place in humans at the 4-cell stage and between the 4-cell and the 8-cell stage of cleavage, respectively. Among these factors, genetic ones and epigenetic ones can be distinguished.

Sperm genetic factors affecting embryo developmental potential can be inherited or acquired. As to the inherited factors, there are a number of chromosomal abnormalities and gene deletions or mutations that impact embryo quality. Notably, many single-gene mutations or expression changes and their respective impacts on sperm function (Table 1) were reviewed recently [74].

While many of these abnormalities are incompatible with full sperm development or fertilizing ability, and are thus excluded from transmission to embryos via natural fertilization, these barriers can now be partly circumvented with the use of micromanipulation-assisted IVF technologies, such as ICSI and ROSI [4,5,6]. In fact, the de novo chromosomal abnormality rate in pre- and postnatal karyotypes of ICSI offspring was shown to be higher than in the general population and related to fathers’ sperm parameters [75]. As to ROSI, the number of analyzable cases is still too low to be assessed.

Sperm DNA fragmentation is the most extensively studied acquired genetic factor related to human embryo dysfunction. Originating mainly from apoptosis (programmed cell death) during early stages of spermatogenesis and oxidative stress and defective chromatin packaging during late spermatogenesis (spermiogenesis), DNA breaks can concern a single DNA strand (oxidative stress, improper chromatin packaging) or both of them (apoptosis) [76].

**Figure 6:**
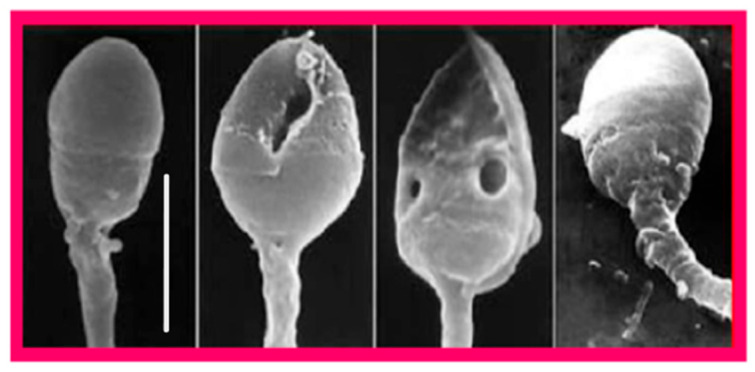
Scanning electron micrographs of human spermatozoa showing apparently normal chromatin condensation (left and right ones) and condensation defects revealed by the presence of intranuclear vacuoles (the two in the center). Scale bar = 5 μm. From Tesarik [77]. Creative Commons Attribution Licence.

Since sperm DNA is protected against insults by its association with protamins in the highly compact chromatin structure, there is a close association of its fragmentation with defective chromatin packaging as revealed by scanning electron microscopy (Figure 6) [77]. Even though, unlike oocytes, spermatozoa are incapable of repairing their own DNA damage, it can be repaired after fertilization in zygotes using maternal DNA repair mechanisms both in mice [78] and humans [79]. General features of DNA damage repair mechanisms and their activity in human zygotes and embryos have been reviewed recently [80]. This DNA repair capacity is limited and likely to decline with female age, and unrepaired DNA damage can disrupt further development of the zygote, potentially leading to pregnancy loss, birth defects, and increased risk of certain diseases, including cancer [81]. Likewise, errors of zygotic repair of sperm-derived DNA can be as destructive as the DNA damage itself, causing mis-rejoining of DNA fragments, chromosomal rearrangements, and the formation of acentric fragments [81].

Another genetic factor which may affect human embryo viability and function is sperm aneuploidy, which results from errors of chromosome synapsis during spermatogenesis, mainly concerns chromosomes X and Y, and is more frequent in spermatozoa surgically retrieved from men with nonobstructive azoospermia [82] or severe oligozoospermia [83]. Aneuploid spermatozoa are capable of fertilizing the oocyte, leading to embryo aneuploidy [84], and this situation is related to lower implantation and pregnancy rates and higher abortion rates after embryo transfer [85].

Epigenetic factors that affect the viability and function of human embryos by acting on paternal DNA, especially in the context of micromanipulation-assisted fertilization, have been extensively reviewed [26,86]. In this section, only those factors acting directly on sperm DNA structure are dealt with. Other epigenetic issues, related to the action of sperm oocyte-activating factor and centriole, were covered in other parts of this article (Section 2 and Section 3, respectively), and those related to the expression of small non-coding RNA are included in Section 5. Traditionally, it was believed that all epigenetic marks, including DNA methylation, histone acetylation status, and small RNAs, are completely erased and subsequently reset during germline reprogramming [87]. In mammals, these events take place both in the germline and in the zygote immediately after fertilization [88]. However, it is now known that this reset is not complete, and some sperm-inherited regions can escape reprogramming to impact functional changes in the pre- and postimplantation embryo development via mechanisms that implicate transcription factors, chromatin organization, and transposable elements [89].

A recent study reported a positive correlation of good embryo quality in human IVF with Histone H3 Lysine 27 trimethylation (H3K27me3) mark, whereas H3K4me3 and H3K4me2 marks were correlated negatively with fertilization rate [90]. During mammalian preimplantation development, H3K27me3 is catalyzed by proteins of the polycomb group, an evolutionally conserved set of long-term transcriptional repressors, and is involved in silencing of gene expression before zygotic gene activation (ZGA) [91]. In human germinal/vesicle oocytes, H3K27me3 was shown to be selectively deposited in promoters of developmental genes and partially methylated domains, and it was strikingly absent in human embryos at ZGA (8-cell) [92], indicating a comprehensive erasure of this histone modification on both parental genomes [93]. A list of aberrantly methylated genes and their related sperm abnormalities has been published recently [94]. The concept of epigenetic modifications of sperm-derived DNA and associated proteins as factors influencing embryo viability and function is thus emerging and represents a challenge for future focused research.

### 4.2. Clinical Resolution

#### 4.2.1. Diagnostic Methods

With the exception of sperm DNA fragmentation, for which various tests are currently available [95], clinically confirmed specific tests for the other sperm DNA issues are still lacking.

#### 4.2.2. Treatment Options

Treatment options to be envisaged in cases of sperm DNA fragmentation have been reviewed recently [96]; they involve treatment of comorbidities, if suspected to be the cause, in vivo therapies given to affected men, and in vitro sperm selection techniques. Specifically, patient-tailored use of oral antibiotics and anti-inflammatory agents to control semen infection [97], hormonal substitution (gonadotropins, selective estrogen receptor modulators, and aromatase inhibitors) [98], oral administration of antioxidant vitamins [99,100], in vitro selection of living spermatozoa with morphologically intact chromatin by high-magnification ICSI (IMSI) [101], and the recourse to testicular spermatozoa as the ultimate measure when all the above fail [102], were demonstrated be of help in cases of excessive sperm DNA fragmentation.

No specific treatments for sperm DNA issues other than fragmentation have been suggested yet. However, because most of these issues emerged in the era of ICSI, it can be speculated that oocyte vestments (cumulus oophorus and zona pellucida), that have to be negotiated by the spermatozoon before it can fertilize naturally, might exert a barrier effect which could selectively prevent spermatozoa carrying different genetic and epigenetic abnormalities from entering [16]. If this hypothesis is confirmed, methods for the selection of spermatozoa for ICSI, based on their affinity to the zona pellucida, will probably be introduced to future clinical IVF practice. Alternatively, when spermatozoa from a given patient are capable of penetrating into oocytes by their proper means, conventional IVF, potentially enhanced by sperm pretreatment with pentoxifylline [103,104], might be used instead of ICSI. Moreover, similar to sperm DNA fragmentation, abnormal histone methylation patterns are also significantly correlated with the presence of intranuclear vacuoles, suggesting that the selection of normal spermatozoa without vacuoles and the deselection of spermatozoa with vacuoles appear to be epigenetically favorable to embryo development and safe offspring [105].

## 5. Sperm RNA

### 5.1. Molecular Basis

The importance of RNA delivered to the oocyte at fertilization (large and small, coding and non-coding RNAs) for embryo development has long been underestimated [106,107], and it is only recently that this subject has received adequate attention, although there still remain many unanswered question as to the underlying mechanisms [89,108,109]. In the mouse model it was shown that, in addition to RNAs synthesized during spermatogenesis, some RNA species are acquired by spermatozoa as they migrate through the male reproductive tract, specifically during their epididymal transit [110,111], and a similar traffic was also reported in idiopathic infertile men undergoing fertility treatment [108]. It was also suggested that particular RNAs may be selectively delivered to spermatozoa through epididymosomes in response to environmental factors [112].

Among the RNA species introduced to the oocyte by the fertilizing spermatozoon, small non-coding RNAs, such as tRNA-derived small RNAs, small interfering RNAs, ribosomal RNA-derived small RNAs, microRNAs, small nuclear RNAs and PIWI-interacting RNAs, modulate embryo development and offspring phenotype, and the resulting modifications may be transgenerationally inherited [94,113]. Most of the effects of these RNAs are related to epigenetic modifications of embryonic gene activity [109], while sperm-borne mRNAs have potential roles in zygote genome activation and epigenetic inheritance [94]. In addition, sperm RNAs were also shown to take part in other essential early events in the embryo, such as chromatin remodeling, nuclear programming, genome activation, and transposable element activity [94,108,112,114,115].

In humans, several hundreds of RNA elements (exon-sized sequences in RNA molecules that can affect gene expression), including microRNAs, transfer RNA-derived small RNA, and small non-coding RNAs, were shown to be significantly associated with blastocyst development, and some of them were closely linked to genes involved in critical developmental processes, such as mitotic spindle formation and specification of ectoderm and mesoderm cell lineages [112]. Similar to the mouse, environmental exposures affect human sperm RNA [116], mainly acting on sperm microRNAs [89], and the presence or absence of specific RNA elements is positively or negatively correlated with idiopathic male infertility [117,118]. On the whole, sperm RNAs are increasingly considered potential markers of sperm-derived embryo dysfunction and therapeutic targets, although the mechanisms of their actions in embryos are still only partly understood.

### 5.2. Clinical Resolution

#### 5.2.1. Diagnostic Methods

Assays for sperm RNAs relevant to embryonic development are emerging only recently and are based on next-generation sequencing to profile RNA extracted from the patients’ spermatozoa [108,118]. Among different types of RNA studied so far by next-generation transcriptome sequencing [119], three microRNAs (hsa-miR-9-3p, hsa-miR-30b-5p, and hsa-miR-122-5p) had the highest potential as biomarkers of male fertility and sperm quality [120].

#### 5.2.2. Treatment Options

No specific treatment for sperm RNA issues is yet available. However, as discussed in Section 4.2.2. dealing with sperm DNA, making use of the natural selection of the most competent spermatozoa in the conventional IVF setting may be envisaged where sperm movement, binding to, and penetration of the zona pellucida are compatible with the use of this technique.

## 6. Sperm Proteins

A deep analysis of the sperm proteome revealed several hundred proteins with known roles in the process of fertilization, including those composing the sperm centrioles (see Section 3.1. of this article), and those with other roles in early embryo development [121].

### 6.1. Molecular Basis

A recent study identified 560 sperm proteins involved in modulating gene expression by regulation of transcription, DNA methylation, histone post-translational modifications, and non-coding RNA biogenesis [121]. Other sperm proteins with a known function, such as those involved in sperm DNA protamination (mainly protamin-2, transitional proteins and histone acetyltransferases) and antioxidant capacity (such as superoxide dismutase type 1 and peroxiredoxin 5) were shown to be essential for the preservation of sperm DNA integrity in the mouse model [122].

### 6.2. Clinical Resolution

#### 6.2.1. Diagnostic Methods

Recent studies resumed the current possibilities of assessing protein markers of sperm functions by proteomics and identified the proteins that could be potential targets for further basic and clinical studies [121,122,123].

#### 6.2.2. Treatment Options

Apart from the treatments for the functional consequences of specific sperm protein deficiencies, including sperm oocyte-activating factor (Section 2.2.2), centrioles (Section 3.2.2), and sperm DNA epigenetic reprogramming (Section 4.2.2), some reports suggest that the overall sperm quality can be improved by appropriate diet [124] and the ingestion of nutrients and dietary supplements [125].

## 7. General Considerations of Gamete Complementarity and Individual Susceptibility

Despite the fact that the present review focuses on sperm-derived factors affecting embryo viability and developmental potential, it needs to be kept in mind that such factors act on specific oocyte and embryo targets, which may be themselves responsible for the observed dysfunction rather than their sperm counterparts. Additionally, the above interactions take place in each individual’s susceptibility background and are influenced by a variety of other factors, such as those related to lifestyle, environmental exposures, metabolic disorders, and abnormal ion channel or receptor activities. Though not dealt with in this article, these interactions need to be fully considered and included in the research outlook and comprehensive clinical management.

## 8. Conclusions

In addition to transmitting the male genome, sperm factors are currently known to play other essential roles in human embryo development. These factors include sperm oocyte-activating factor, centrioles, DNA, RNA, and proteins, with each of them affecting different embryo developmental characteristics. The nature of perturbations detected can thus call attention to the factor most likely to be involved. Abnormalities of sperm-induced oocyte activation can entail total fertilization failure, atypical pronuclear development, de novo chromosomal numerical abnormalities, and recurrent failures of embryo cleavage, implantation, and postimplantation development. Defective function of sperm centrioles causes pronuclear abnormalities, impairs embryo morphology (fragmentation), and leads to implantation failure and miscarriage. Sperm DNA and RNA issues can be at the origin of recurrent pregnancy loss and birth defects.

The sperm origin of any of the above abnormalities is more likely in couples in whom no problems relative to the female reproductive health can be detected. However, the presence of the female factors does not exclude superimposition of male factors, which should, thus, never be forgotten. Except for sperm DNA fragmentation, the diagnosis of the other potential sperm factors is more difficult. The dysfunction of sperm oocyte-activating factor (PLCζ) can be assessed indirectly, by heterologous ICSI with mouse or hamster oocytes, or directly by the evaluation of PLCζ abundance and distribution in spermatozoa or related ultrastructural anomalies. Heterologous ICSI (rabbit or bovine oocytes) can also serve to evaluate the function of sperm centrioles. Abnormalities in sperm DNA (other than fragmentation) can be approached indirectly by assessing the presence of RNAs and proteins involved in epigenetic reprogramming.

Available treatments of sperm-derived embryo dysfunction include assisted oocyte activation (for oocyte-activating factor), oral antioxidants, treatment of comorbidities, and high-magnification selection of spermatozoa to be used in ICSI (for sperm DNA fragmentation). No clinically confirmed specific treatments yet exist for other DNA anomalies and issues of RNA and proteins, but the use of conventional IVF instead of ICSI, to benefit from natural sperm selection, might be considered where feasible.

Difficulties of distinction between sperm-derived and oocyte-derived factors of embryo demise, which may act in synchrony, are the main limitations of the correct interpretation of observational data leading to appropriate treatment decision-making. Further development of quick and easy diagnostic tests is, thus, an important direction for future research.

## Figures and Tables

**Table 1:** Genes in which mutations or gene expression changes were identified with the respective infertility phenotype associated.

Infertility Phenotype	Genes
Asthenospermia	*AKAP3*, *AKAP4*, *AXDND1*, *CATSPER1*, *CATSPER2*, *CATSPER3*, *CATSPER4*, *CCDC103*, *CCDC40*, *CFAP43*, *CFAP44*, *CFAP70*, *COPS7A*, *CRHR1*, *CUL3*, *DEFB126*, *DNAAF1*, *DNAAF6*, *DNAH6*, *DNAH11*, *DNAH17*, *DNAH5*, *DNAH8*, *DNAH9*, *DNAI1*, *DNAJB13*, *DNHD1*, *DRC1*, *HIP1*, *HTX11*, *INSL6 IQCG*, *IQUB*, *KLHL7*, *KRT34*, *LRRC6*, *MT-C03*, *NEDD4*, *NSUN7*, *QRICH2*, *RSPH3*, *RSPH6A*, *SEPTIN4*, *SLC26A8*, *SPAG17*, *SPATA33*, *TEKT2*, *ZMYND10*
Multiple morphological anomalies of the flagella (MMAF)	*BRWD1*, *CCDC34*, *CCDC39*, *CEP135*, *CFAP251*, *CFAP58*, *CFAP61*, *CFAP69*, *CFAP74*, *DNAH1*, *DNAH10*, *DNAH17*, *DNAH2*, *DNAH5*, *DNAH6*, *DNAH7*, *DNAH8*, *DZIP1*, *DZP1*, *FSIP2*, *MAATS1*, *ODF2*, *QRICH2*, *SPAG6*, *SPATA16*, *SPEF2*, *TTC21A*, *TTC29*, *WDR19*, *WDR66*
Nonobstructive azoospermia	*AR*, *ABLIM1*, *AHRR*, *ART3*, *ATM*, *AZFa*, *AZFb*, *AZFc*, *BCL2*, *BPDY2*, *BPY2*, *CCDC34*, *CDC42BPA*, *CDY2A*, *DAZ1*, *DBX3Y*, *DMC1*, *DMRT1*, *DNMT3B*, *EPSTI1*, *ETV5*, *FANCM*, *GNAO1*, *HLA-DRA*, *HSF2*, *HSFY1*, *KLHL10*, *M1AP*, *MCM8*, *MEIOB*, *MLH3*, *MSMB*, *MTHFR*, *NANOS1*, *NPAS2*, *NR5A1*, *PACRG*, *PIWIL2*, *PNLDC1*, *PYGO2*, *RBMX*, *RBYMIAI*, *REC8*, *SIRPG*, *SOHLH1*, *SOX5*, *SPINK2*, *SRSF6*, *STAG3*, *STX2*, *SYCE1*, *SYCE1L*, *SYCP3*, *TAF4B*, *TDRD9*, *TEX11*, *TEX14*, *TEX15*, *USP9Y*, *WT1*, *XRCC2*, *ZMYND15*
Obstructive azoospermia	*ADGRG2*, *CFTR*
Oligozoospermia	*AXDND1*, *DAZ1*, *DAZ2*, *DICER1*, *DNMT1*, *EPHX2*, *GSTM1*, *GSTT1*, *KIT*, *KITLG*, *NR0B1*, *NR5A1*, *OR2W3*, *PARP1*, *PIWIL3*, *PIWIL4*, *PLK4*, *PON1*, *PON2*, *PRM1*, *PSAT1*, *SIRPA*, *SOX6*, *USP8*, *ZMYND15*
Teratozoospermia	*AURKC*, *BSCL2*, *CCIN*, *CCDC90B*, *CCDC91*, *DPY19L2*, *SPATA20*, *SPA17*, *CYP1A1*, *FBXO43*, *PPP2R3C*, *SEPTIN12*, *ZPBP*, *DPY19L2*, *PICK1*, *SPATA16*, *SEPTIN4*

From Pereira and Sousa 2023 [74]. © 2023 by the authors. Creative Commons Attribution Licence.
